# Differential aspects of familiar people recognition disorders in right and left variants of semantic dementia: A critical review

**DOI:** 10.1111/jnp.70057

**Published:** 2026-06-05

**Authors:** Guido Gainotti

**Affiliations:** ^1^ Institute of Neurology Università Cattolica del Sacro Cuore, Fondazione Policlinico A. Gemelli, IRCCS Rome Italy

**Keywords:** familiar people recognition disorders, familiarity feelings, verbal and non‐verbal semantic disorders, right and left variants of semantic dementia, right anterior temporal lobes

## Abstract

The aim of the present review will consist of discussing the different forms of familiar people recognition disorders observed in the right and left variants of semantic dementia. The review will start with a summary of the main cognitive models of familiar people recognition and of the neuroanatomical correlates of the processing stages proposed by these models. In this section, the role played by ‘familiarity feelings’ and by the properly ‘semantic’ aspects of familiar people recognition in the recognition of familiar people will be taken separately into account. The different roles played by the right and left anterior temporal lobes in each of these components of the system will also be stressed. In the second part of the review, I will discuss results obtained by patients with right and left variants of semantic dementia on tasks assessing ‘familiarity feelings’ and the distinction between general and social knowledge, reaching the following conclusions: (a) recognition disorders shown by patients with a right variant of semantic dementia are due to more to the disruption of pre‐semantic mechanisms allowing the automatic generation of familiarity feelings than to properly semantic defects; (b) the right anterior temporal lobe (ATL) supports more the concrete than the abstract components of ‘social knowledge’; (c) the right ATL can store only information that can be represented in a concrete pictorial format whereas the left ATL mainly stores information that can be represented only through abstract linguistic propositions (240).

## INTRODUCTION

According to many studies of semantic dementia, (SD) (e.g. Josephs et al., 2009; Snowden et al., 2004; Ulugut et al., 2024; Ulugut Erkoyun et al., 2020; Yadollahikhales et al., 2024; Younes et al., 2022), familiar people recognition disorders are among the most frequent and typical landmarks of fronto‐temporal degeneration (FTD). Most of these studies have also noted that people recognition disorders take a different form in the left and right variants of this disease. This problem was initially raised by the observation of individual patients with predominantly right temporal atrophy who presented a disproportionate impairment in recognition of familiar people (e.g. Evans et al., 1995; Gainotti et al., 2003; Joubert et al., 2003) In these patients, face recognition disorders (prosopagnosia) were usually on the foreground and voice recognition disorders (phonagnosia) were also often observed, but proper name recognition was relatively spared. Systematic reviews of these single‐case studies can be found in Gainotti (2013).

These seminal observations were confirmed by investigations studying face recognition disorders on groups of SD patients (e.g. Josephs et al., 2009; Luzzi et al., 2017; Snowden et al., 2004; Yadollahikhales et al., 2024; Younes et al., 2022) or of patients with other diseases asymmetrically involving the ATLs (e.g. Borghesani et al., 2019; Piccininni et al., 2023). However, some of these more controlled studies also showed that, due to the complexity of this task, the left/verbal versus right/non‐verbal dichotomy is too limited to account for the complexity of familiar people recognition disorders. In human studies, where clinical and experimental investigations have mainly focused on the analysis of face recognition processes, different authors (e.g. Bruce & Young, 1986; Burton et al., 1990; Ellis et al., 1997) have proposed cognitive maps, based both on computational models and on neuroanatomical data that aimed to explain: (a) how the familiar people identification process is achieved within each recognition modality, (b) how these modalities interact and (c) how the semantic (biographic) information characterizing each person are accessed and organized. Due to the importance of these subjects, I thought that a quick reminder of the models proposed by Bruce and Young (1986) and by Ellis et al. (1997) and of their neuroanatomical correlates could be useful to understand the further sections of this revue. Only after this brief theoretical introduction, I will pass to discuss their implications for familiar people recognition disorders in the right and left variants of SD.

At the end of this introductory section, I would also shortly say how this review differs from other papers on closely related topics, stressing in particular two points. The first is that, since the differential aspects that distinguish the right from the left variants of SD are mainly observed in the first stages of the degenerative process, my attention will be focused on these early stages, in which the lesion selectively affects the right ATL. The second is that, since the role of modality‐specific ‘familiarity feelings’ in the recognition of familiar people is often neglected, whereas much debated are the distinctions between ‘general vs social knowledge’ and between ‘verbal and non‐verbal semantic representations’, all these points will receive equal attention in the discussion of the mechanisms underlying the different familiar people recognition disorders observed in the right and left variants of SD.

## COGNITIVE MODELS OF FAMILIAR PEOPLE RECOGNITION AND NEUROANATOMICAL CORRELATES OF THE VARIOUS STAGES OF THESE MODELS

According to Bruce and Young (1986) the input of some lower level perceptual processes, mapped onto specific recognition units for faces (FRUs) and voices (VRUs), converge into person‐identity nodes (PINs), allowing access to the semantic information of the corresponding known person. Relying on this seminal model, Ellis et al. (1997) postulated the existence of two processing modules for faces and voices that worked in parallel and included three steps. The first step should allow perceptual processing of visual and auditory inputs leading to their ‘structural encoding’, whereas the second step should be characterized by the activation of modality‐specific ‘recognition units’ for face (FRU) and voice (VRU), where the corresponding familiarity feelings should be generated. The final step should lead to the convergence of these two pathways into a ‘person identity node’ (PIN), storing semantic information about the identified person. A neurobiological approach to the difference between the perceptual and the post‐perceptual levels of familiar people recognition has been made by Haxby et al. (2000), who have subdivided the face recognition network into a ‘core’ system which is responsible for primarily perceptual processing and an ‘extended’ network underpinning further cognitive aspects of face processing and access to person semantic information. The ‘core’ face (and voice) processing systems should comprise posterior occipital and temporal regions, such as the fusiform face area, the occipital face area, and the posterior superior temporal sulcus, whereas the ‘extended’ network should comprise structures located along a posterior‐to‐anterior axis, where responses become increasingly tuned to more complex feature combinations. This extended network should end with semantic processing structures within the ATL, where face and voice‐specific representations could be stored respectively on the ventral and dorsal parts of the ATLs (Olson et al., 2013). The large majority of studies which have investigated familiar people recognition disorders in the right and left variants of SD has been based on this distinction, which has, however, usually not taken into account an important intermediary mechanism between perceptual and semantic processing, namely, the automatic evocation of ‘familiarity feelings’ when the stimulus represents a person known to the patient.

### The role of ‘familiarity feelings’ in the recognition of familiar people

This mechanism, that plays a key role in the recognition of familiar people and had been included in their models by Bruce and Young (1986) and Ellis et al. (1997), is linked to the automatic generation of a ‘familiarity feeling’, that is triggered when an incoming perceptual pattern matches a stored representation of the same stimulus. According to many authors (e.g. Gobbini et al., 2004; Gobbini & Haxby, 2007) the automatic generation of these ‘familiarity feelings’ is, at least in part, due to the impact of emotional factors, related to the personal significance of the known person. Studies conducted on healthy subjects and on patients with unilateral brain damage (Gainotti, 2007, 2011) have also shown that the generation of familiarity feelings prevails, on the right side when the evoking stimulus is a familiar face or voice and on the left side when the evoking stimulus is a familiar personal name. Therefore, since a relation exists between the verbal or non‐verbal nature of the evoking stimulus and the side where the familiarity feeling is generated, these feelings could also play a different role in the production of verbal and non‐verbal disorders of people recognition in right and left variants of SD.

### The properly ‘semantic’ aspects of familiar people recognition: The distinctions between ‘general versus social knowledge’ and between ‘verbal vs non‐verbal’ representations

A second, even more important source of controversies in these studies has regarded the properly semantic aspects of these disorders because two important distinctions have been proposed on this subject. The first concerned the content of semantic memory, distinguishing the ‘social semantics’ from the ‘general semantics’, whereas the second regarded the format of these representations, distinguishing the ‘verbal’ (proper name) from the non‐verbal (face and voice) modalities of familiar people recognition. As a matter of fact, even if the hypothesis of a unitary single semantic network is strongly defended by the influential ‘hub and spoke’ model, proposed by Lambon Ralph et al. (2017), several authors have proposed that this network may be fragmented with respect both to the content and to the format of the stored knowledge.

#### The distinction between ‘general knowledge’ and ‘social knowledge’

As for the first point, earliest cognitive models of face identification and people's name processing (Bruce & Young, 1986; Burton et al., 1990) had postulated that distinct pathways might be involved in the access to general semantic knowledge and person‐specific knowledge. In more recent times, several authors (e.g. Olson et al. 2007, 2013) have suggested that memory for people and their relationships, along with memory for social behaviours, may constitute a specific type of semantic memory labelled social knowledge and that portions of the ATLs may play a critical role in representing and retrieving social knowledge, three main sources of evidence, linked to anatomical research (e.g. Ding et al., 2009) ablation studies in animals Olson et al. (2007), and functional neuroimaging (e.g. Thye et al., 2024) have been reported by Olson et al. (2013) as evidence of the links between ATLs and social knowledge. The term social semantics was thus proposed to group together all the concrete (person‐specific) and abstract (social rules) knowledge required for social interactions even if the relations between these sources of knowledge and the ATLs did not necessarily point to the existence of a specific social semantic system including both concrete items (representation of individual persons) and abstract concepts (social norms).

#### The distinction between ‘verbal’ and ‘non‐verbal’ representations

As for the second point, concerning the distinction between ‘verbal’ and ‘non‐verbal’ representations, the hypothesis assuming that semantic information may be stored in the brain in two different (verbal vs. non‐verbal) formats was documented by Snowden et al. (2004) who assessed knowledge of famous faces and names in SD patients, with right or left prevalence of the brain atrophy. These authors showed that patients with left ATL atrophy identified faces better than names, whereas patients with a more severe right ATL degeneration showed the opposite pattern and that a better identification of famous faces was associated with superior performance on the picture, compared with the word versions of the Pyramids and Palm trees test. These data were confirmed by Gainotti (2012), who reviewed all the published cases of patients with a prevalent damage to the right or left ATLs, in whom person recognition disorders were on the foreground, showing that the loss of personal and general semantics is prevalent from faces (and object pictures) when the right ATL is damaged and from names when the anterior parts of the left ATL are selectively affected.

## IMPLICATIONS OF DATA CONCERNING ‘FAMILIARITY FEELINGS’ AND THE DISTINCTION BETWEEN GENERAL AND SOCIAL KNOWLEDGE TO UNDERSTAND PEOPLE RECOGNITION DISORDERS IN THE RIGHT AND LEFT VARIANTS OF SEMANTIC DEMENTIA

Data concerning ‘familiarity feelings’ and the distinction between general and social knowledge could allow to understand different aspects of familiar people recognition disorders for two different sets of reasons. The first set concerns the automatic generation of a ‘familiarity feeling’ when a perceptual pattern matches the stored representation of a face, voice or personal name, and is linked to the fact that these feelings: (a) are modality‐specific; (b) prevail on the right side when the stimulus is a face or voice and on the left side when it is a personal name (Gainotti, 2007, 2011) and (c) could contribute to the search within the semantic system of the biographic information corresponding to the ‘familiar’ stimulus. The second set of reasons concerns the distinction between social and general semantics and underlines the importance of investigating if all aspects of social knowledge are equally disrupted in patients with a right or left‐sided variant of SD or if only some components are affected. Neuropsychological investigations have, therefore, tried to disentangle, within the person recognition disorders shown by patients affected by ATL lesions, the role played respectively by a defective generation of appropriate familiarity feelings and by a defective retrieval of the appropriate semantic information. These two groups of investigations, linked to different aspects of people recognition disorders, will be taken separately into account in the next section of this review.

### Neuropsychological studies which have tried to clarify the role of defective familiarity feelings and of properly semantic recognition disorders in patients affected by a right or left variant of SD

Studies aiming to clarify the role of defective familiarity feelings on person recognition disorders have administered to groups of patients affected by a right or left variant of SD (or of other diseases asymmetrically involving the ATLs) tasks of familiar people recognition that allowed to distinguish the impairment of different levels of face, voice, and name processing. These levels concerned respectively: (a) the purely perceptual discrimination; (b) the distinction between familiar and unfamiliar items; (c) the retrieval of semantic information about the known person; (d) the name of this person.

Five studies of this kind have been conducted by Papagno et al. (2018), Snowden et al. (2018), Borghesani et al. (2019), Piccininni et al. (2023) and Yadollahikhales et al. (2024). The main characteristics (patients, methodology, and results) of these studies have been reported in Table 1.

**TABLE 1:** Characteristics of neuropsychological studies which have tried to disentangle disorders affecting different levels of person recognition in patients affected by a right or left variant of SD.

Authors	Papagno et al. (2018)
Patients	Twenty‐nine patients who underwent surgery for a temporal glioma, either in the left (16 patients) or right (13 patients) hemisphere
Modalities of person recognition investigated	Perceptual and post‐perceptual disorders of voices recognition were assessed with standardized tests of unknown voice discrimination (UVD) and of famous voice recognition (VO‐REC), which included tasks of familiarity evaluation, semantic identification and naming of famous voices
Results	Patients with right temporal gliomas were significantly more impaired in voices discrimination and produced more false alarms (indicative of impaired familiarity) than patients with a left glioma, whereas the latter were significantly more impaired in name retrieval than patients with a right temporal glioma
Authors	Borghesani et al. (2019)
Patients	Data from a large sample of 105 patients with neurodegenerative disorders, including 43 SD patients, were analysed to identify the neuroanatomical substrate of three different steps of famous face processing.
Modalities of person recognition investigated	Famous face processing was assessed using the UCSF Famous Faces Battery, which comprises three different tasks of Famous Face Familiarity Judgement, Famous Face Semantic Association and Famous Face Confrontation Naming
Results	A significant difference was found between right ATL damage (associated with deficits in familiarity judgements) and left ATL damage (associated with deficits in naming and semantic/biographical information retrieval).
Authors	Piccininni et al. (2023)
Patients	Fourty‐three patients, affected by semantic dementia (*n* = 11) or by temporal tumours (*n* = 32), showing an involvement of the right or left anterior temporal lobes were investigated
Modalities of person recognition investigated	Patients were given standardized tests of unknown face and voice discrimination (FaVoD) and the ‘Famous People Recognition Battery’ (FPRB), which included tasks of familiarity evaluation, semantic identification and naming of famous faces and voices
Results	At the familiarity level, patients with right ATL lesions showed a greater impairment of on the non‐verbal (face and voice) recognition modalities whereas patients with left ATL lesions were more impaired on name familiarity. At the semantic level no difference was found between the two hemispheric groups whereas at on confrontation naming of faces and voices the worst results were obtained by left‐sided patients
Authors	Yadollahikhales et al. (2024)
Patients	Seventy‐four SD patients (54 with predominant left and 20 with predominant right ATL atrophy) and 36 healthy controls (HC) were examined
Modalities of person recognition investigated	They underwent a perceptual face processing test (Benton facial recognition test‐short version; BFRT‐S), and semantic face processing tests (UCSF Famous people battery – Recognition, Naming, Semantic associations – pictures and words subtests), as well as structural magnetic resonance imaging (MRI)
Results	Both right and left SD patients were impaired on all semantic face processing tests, with right‐sided patients performing significantly lower on the famous faces' recognition task, and left‐sided patients performing significantly lower on the naming task despite their spared performance on the familiarity judgement task

Results of these investigations have been consistent across modalities, both in studies involving voice (Papagno et al., 2018; Piccininni et al., 2023) and in those concerning face recognition (Borghesani et al., 2019; Piccininni et al., 2023; Snowden et al., 2018; Yadollahikhales et al., 2024). Face and voice perceptual discriminations were, indeed, usually unimpaired, irrespective of the side of predominant atrophy, whereas interhemispheric differences emerged at the post‐perceptual level. In particular, face and voice familiarity feelings were more impaired in patients with right‐sided lesions (Borghesani et al., 2019; Papagno et al., 2018; Piccininni et al., 2023; Yadollahikhales et al., 2024), whereas confrontation naming (of the same stimuli) was mainly affected by left ATL damage (Borghesani et al., 2019; Papagno et al., 2018; Piccininni et al., 2023; Yadollahikhales et al., 2024). Furthermore, in the Snowden et al. (2018) study, a consistency was found between results obtained by right‐sided patients on tests of face and name familiarity judgement and those obtained by the same patients on the visual and verbal versions of a task of general semantics.

Less clear was the relation between side of ATL atrophy and retrieval of face and voice related properly semantic information, because in the two studies, by Borghesani et al. (2019) and Piccininni et al. (2023), that specifically investigated this problem, no relation was documented between this level of impairment and right ATL damage. Piccininni et al. (2023) found no difference on this subject between patients affected by right and left ATL lesions, and Borghesani et al. (2019) found an association between semantic/biographical information retrieval and prevalent lesion of the left ATL. Results only in part consistent with the hypothesis assuming that only pre‐semantic (but not properly semantic) disorders of person recognition may be observed in patients with a right variant of SD have been obtained by Joubert et al. (2006), who, in a well conducted investigation, have found that right ATL patients can show a loss of semantic person knowledge, associated with defects of familiarity feelings. These authors investigated familiarity, semantic knowledge and naming of familiar people through face, voice, and name in three patients with the right variant of frontotemporal dementia and a group of 20 control subjects. Patients with the right variant of SD were significantly impaired on familiarity for faces and voices (but not for names) and were unable to provide accurate semantic information about famous people irrespectively of the modality of presentation. These findings will, therefore, be reconsidered in the Discussion section.

### Are familiar people recognition disorders of patients affected by SD due to a more general disruption of ‘social knowledge’?

The hypothesis assumes that familiar people recognition disorders of patients affected by SD may be due to a more general disruption of ‘social knowledge’ was first advanced by Thompson et al. (2004), who reported two SD patients (JP and MA), who exhibited opposite patterns of performances for person‐specific and, respectively for general semantic knowledge. Patient JP, with predominantly right temporal atrophy showed severely impaired person‐specific semantics, with relative preservation of knowledge about objects and animals, while patient MA, with predominantly left temporal atrophy exhibited the opposite pattern of performance (good knowledge of people in the context of impoverished general semantics). The Thompson et al. (2004) hypothesis was, however, rejected by Snowden et al. (2012) who showed that in SD patients, right‐sided atrophy is associated with greater impairment for faces and general visual knowledge, whereas left‐sided atrophy is associated with greater impairment for names and general verbal knowledge. Other investigations conducted in healthy subjects or in brain‐damaged patients supported the hypothesis of a general relation between social knowledge and the right ATL and between other categories of knowledge and the left ATL. Thus, Zahn et al. (2009) identified in a functional MRI study, a right superior ATL region showing selective activation for ‘social concepts’ as compared with concepts describing non‐social animal behaviour. Irish et al. (2014) investigated the neural correlates of theory of mind performance in patients with SD and showed that theory of mind impairments correlated significantly with atrophy in right ATLs. Other investigations provided, however, more conflicting results about the relations between social knowledge and right ATL and general knowledge and left ATL. Thus, Zahn et al. (2007) showed that in healthy participants. activation associated with socially related words (e.g. polite) versus non‐social words (e.g. nutritious) was localized to the right superior ATL, but a direct replication of the Zahn et al. (2007) task by Ross and Olson (2010) found greater activation for social than non‐social words in the left rather than in the right superior ATL, suggesting that both ATLs may play a role in social concepts. Pobric et al. (2016) investigated direct evidence for the functional relevance of the superior ATL in processing social concepts, using converging evidence from non‐invasive brain stimulation and neuropsychology. They demonstrated that the left superior ATL is necessary for processing both social and non‐social abstract concepts, whereas social conceptual processing predominates in the right superior ATL. Binney et al. (2016) tried to evaluate if different types of concepts, especially those with less reliance upon external sensory experience (such as abstract and social concepts), are coded across the graded ATL hub. They showed that both types of concepts engaged a core left hemisphere semantic network, including the ventrolateral ATL. Rice et al. (2018) used data from three studies comparing concrete versus abstract forms of social concepts (i.e. socially relevant abstract words vs. socially relevant concrete words (e.g. famous names) to assess if ATL subregions are engaged for many kinds of social knowledge or only for person‐related knowledge). They showed that a bilateral ventral ATL cluster responded to all categories in all modalities, whereas comparing socially relevant abstract words to socially relevant concrete words (e.g. famous names), they found stronger activation for both kinds of socially relevant concepts in the left temporo‐parietal junction and the posterior MTG. In a more recent study of social–emotional semantics in patients with right temporal degeneration, Younes et al. (2022) have shown that a right predominant ATL degeneration mainly disrupts the neural representations of non‐verbal semantic knowledge for socio‐emotional concepts, resulting in early, prominent deficits in empathy, people recognition, and social behaviour. These authors have interpreted their data according to the Winkielman et al.'s (2018) model of a dynamic grounding of ‘emotion concepts’, which links the realm of the abstract with that of bodily experience and actions. They have, therefore included emotional experience within the realm of social semantic knowledge, reducing the distance between subjective/personal and objective/social components of semantic knowledge. Therefore, it remains to be decided if abstract, conventional social rules and concrete emotional experience belong to the same or to different categories of knowledge and I will return on this point in the Discussion section.

## DISCUSSION

In my opinion, the main results of this review can be summarized as follows:

Clear differences can be found between disorders of familiar people recognition observed in the right and left variants of SD. These differences, however, cannot be fully explained on the basis of global distinctions between ‘general semantics and social semantics’ or between ‘verbal and non‐verbal representations’.

I think, on the contrary, that at least three reasons suggest making distinctions between different components of the terms ‘semantic’, ‘social semantic’ and ‘non‐verbal representations’ that have been repeatedly used in the present review.

The first issue concerns the appropriateness of using the term ‘semantic' to denote all the post‐perceptual person recognition disorders shown by patients with a right variant of SD (or, more in general, by patients with right ATL damage), because data reported in Section Neuropsychological studies which have tried to clarify the role of defective familiarity feelings and of properly semantic recognition disorders in patients affected by a right or left variant of SD indicate that recognition disorders shown by these patients were not due only to properly semantic defects, but also (and perhaps even more) to the disruption of pre‐semantic mechanisms allowing the automatic generation of familiarity feelings for known faces and voices. These disorders should probably be considered as ‘pre‐semantic’ rather than properly semantic, because they were not due to a defective retrieval of specific autobiographic information stored in semantic memory, but to the disruption of preliminary automatic mechanisms, partly linked to emotional (self‐relevance) factors, that allow to distinguish familiar from unfamiliar people. Gainotti (2025) has, in fact, noted that stimuli representing items of other semantic categories do not require a preliminary check of self‐relevance, because the perceptual features of these stimuli are directly matched to those of the corresponding semantic representations. On the contrary, the recognition of face or voice of a known people requires a preliminary check of self‐relevance and the positive result of this relevance check is attested by the automatic generation of a ‘familiarity feeling’. The involvement of emotional factors in the generation of face familiarity feelings was prompted by the Haxby et al.'s (2000) proposal of an extended neural system linking the core visual face areas to regions functionally dedicated to more complete face processing. Gobbini et al. (2004) and Gobbini and Haxby (2007) have, indeed, shown that the emotional components of the face recognition system include the amygdala, insula, and the striatum and the relations between emotional variables, familiarity factors and face and voice recognition have been acknowledged by several authors (e.g. Chauhan et al., 2023; Schweinberger & Soukup, 1998). The stress put in this review on the role played by defective familiarity feelings on the person recognition disorders shown by patients with a right variant of SD does not imply that we attribute to this mechanism all their ‘semantic’ person recognition defects, because Joubert et al. (2006) have shown that right ATL patients can show loss of semantic person knowledge, in addition to defects of familiarity feelings. Even these authors confirmed however: (a) that the loss of familiarity feelings is modality‐specific, because patients with the right variant of SD were significantly impaired on familiarity for faces and voices but not for names and (b) that recognition disorders for faces and voices were due in part to a properly semantic and in part to a pre‐semantic (familiarity) defect. The hypothesis assuming that the prevalence of face and voice recognition disorders in patients with right variants of SD may be at least in part due to the disruption of the automatic generation of familiarity feelings, is also consistent with a more general model of the hemispheric asymmetries that I have reviewed in previous papers (e.g. Gainotti, 2024). This model assumes that the automatic and unconscious operating modalities typical of the emotional system also permeate other activities of the right hemisphere, whereas the propositional and conscious operating modes typical of the cognitive system characterize the left hemisphere.

The second issue concerns the general construct of ‘social knowledge’ and suggests to differentiate, at least in the right hemisphere, the abstract from the concrete forms of this kinds of knowledge, because emotions, subserved by the right hemisphere (Gainotti, 2019) are linked to concrete aspects of social knowledge (such as the perceptual representations of familiar people), that are also processed by this hemisphere. The hypothesis assuming that only in the right ATLs concrete components of ‘social knowledge’ may interact with emotional mechanisms is consistent with the historical development of studies which have suggested a relation between right ATL and social knowledge. As a matter of fact, the first studies which had suggested this relation (e.g. Glosser et al., 2003) had reported an impairment of patients with right ATL damage on concrete social tasks, such as the recognition of familiar/famous people, whereas investigations, that have studied with functional MRI the neural correlates of concrete and abstract forms of social knowledge, have given contrasting results. Some studies have found a right hemisphere bias (Zahn et al., 2007) while others have identified the opposite link (Ross & Olson, 2010), failing to find a clear relation between the abstract aspects of social knowledge and a right ATL activation. Furthermore, authors who have investigated with purely verbal tasks the distinction between abstract and the concrete forms of social knowledge (e.g. Binney et al., 2016; Rice et al., 2018), have found that the activation evoked by these socially relevant verbal concepts was stronger in the left than in the right temporal lobe. In my opinion, dissociations reported in this review are consistent with the hypothesis assuming that the right hemisphere may processes concrete emotional experience about persons, rather than with the alternative hypothesis assuming that it also supports abstract, conventional social aspects of social knowledge. As a matter of fact, according to the social knowledge hypothesis, patients with a right variant of SD should be impaired both on verbal and on non‐verbal aspects of person recognition, since both are part of our social knowledge, but in this review, only the non‐verbal (face and voice) modalities were affected. Furthermore, in these patients, the lesion mainly affected the generation of familiarity feelings, namely, the most concrete and personally relevant aspect of person recognition, whereas relatively spared was the capacity of finding their names, that is an abstract and conventional form of social knowledge. Taken together, these dissociations suggest that the defect of these patients concerns more a concrete emotional format of familiar person recognition than a category of concrete and abstract aspects of social knowledge.

The last problem that requires, in my opinion, some final reflections concerns the relationships between the ‘verbal vs non‐verbal’ representations and the ‘left versus right ATL structures’ underpinning these different representations. This problem stems from the results of studies (reported in Section Neuropsychological studies which have tried to clarify the role of defective familiarity feelings and of properly semantic recognition disorders in patients affected by a right or left variant of SD) that have investigated familiarity evaluation, semantic identification and naming of famous faces and voices in patients with right and left variants of SD. As a matter of fact, in the two studies that took separately into account results obtained on tasks of familiarity evaluation and of properly semantic identification (Borghesani et al., 2019; Piccininni et al., 2023), only results of familiarity evaluation of faces and voices correlated significantly with integrity of the right ATLs, whereas similar correlations were not found in the properly semantic tasks of person identification. In the Piccininni et al. (2023) study no significant difference was found between patients with right and left ATL atrophy on tasks of semantic identification of famous faces and voices and in the Borghesani et al. (2019) study, scores of semantic association of famous faces significantly correlated with grey matter volume in the left, rather than in the right, ATL. Furthermore, if in the Piccininni et al. (2023) study, the lack of interhemispheric differences could be attributed to the verbal questions used to assess the semantic features associated with the famous faces and voices, this could not be the case for the Borghesani et al. (2019) study, because in their ‘Famous Face Semantic Association’ task, patients had been instructed to match two famous faces – among three choices – according to their profession. These results seem to indicate that partly different semantic information are stored in the right and left ATL, because the right ATL can store only information that can be represented in a concrete pictorial format, whereas only the left ATL can store information that can be represented exclusively through abstract linguistic propositions.

**FIGURE 1:**
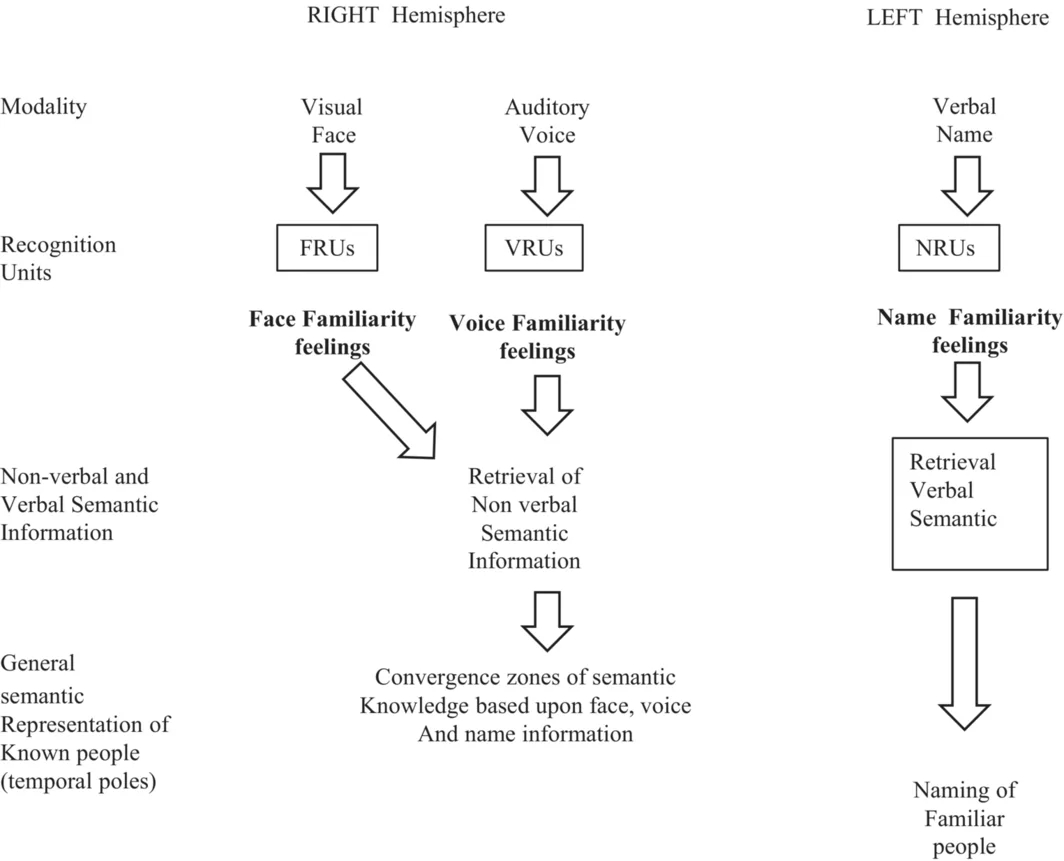
Links that could exist between neuroscientific models of familiar people representation and aspects of the recognition disorders mainly observed in the right and left variants of semantic dementia. On the left side of the schema are represented the components of familiar people recognition (generation of familiarity feelings and semantic information drawn from faces and voices) mainly affected in patients with right ATL damage, whereas on its right side are reported the components (generation of name familiarity feelings and production of the person's name) disrupted in patients with left ATL lesions.

Since I am aware of the complexity of this topic, I have tried to improve its comprehension by summarizing in Figure 1 a schematic representation of the links that could exist between neuroscientific models of familiar people representation and aspects of their recognition disorders mainly observed in the right and left variants of SD.

## CONCLUDING REMARKS

Data reported in this review seem to indicate that post‐perceptual disorders of familiar people recognition observed in the right and left variants of SD can hardly be explained on the basis of simple dichotomies such as the distinctions between ‘verbal and non‐verbal representations’ or between ‘general and social semantics’. These distinctions certainly remain very important, but they should be supplemented by differentiations within each of these general dichotomies trying to take into account the interactions that could exist between the processing of linguistic/abstract and, respectively, of emotional/concrete kinds of knowledge. More in general, results of the present review are not very consistent with strongly ‘unitary’ models of the ‘semantic system’, such as the ‘Semantic Hub’ model (e.g. Lambon Ralph et al., 2017; Rice et al., 2018), because they show that the construction of our knowledge is based not only on abstract general principles, but also on concrete experiential mechanisms, interacting with the emotional system and requiring a non‐unitary treatment of different kinds of knowledge.

## AUTHOR CONTRIBUTIONS

**Guido Gainotti:** Conceptualization; writing – original draft; investigation; funding acquisition; methodology; validation; visualization; writing – review and editing; formal analysis; software; project administration; data curation; supervision; resources.

## CONFLICT OF INTEREST STATEMENT

The author declares no conflicts of interest.

## Data Availability

Data sharing not applicable to this article as no datasets were generated or analysed during the current study.
